# Advances to Electrode Pullback in Cochlear Implant Surgery

**DOI:** 10.1100/2012/126767

**Published:** 2012-10-22

**Authors:** Ingo Todt, Dietmar Basta, Rainer Seidl, Arne Ernst

**Affiliations:** Department of Otolaryngology, Unfallkrankenhaus Berlin, 12683 Berlin, Germany

## Abstract

*Objective*. To observe the intracochlear behavior of a cochlear implant electrode insertion technique (called “pullback”) in temporal bones. *Study Design*. Experimental. *Settings*. Tertiary referral center. *Method*. The change of the intracochlear electrode position was investigated under various conditions of an electrode pullback (*N* = 54) in 9 radiologically, size-estimated temporal bones (TBs). Those TBs were prepared by removal of the cochlear scalar roof to apply digital video capture procedures to monitor the pullback procedures. The digitally captured pictures were analyzed with specific software. *Results*. An optimal pullback of the electrode varied between 1.37 mm and 2.67 mm. While a limited pullback is without risk, an extended pullback bears the risk of removing the electrode tip out of its initial position or out of the cochlea. A correlation between cochlear size and the amount of pullback was not found. *Conclusion*. An initial insertion to the first or the second marker on the electrode followed by a limited pullback of about 1.37 mm to 1.5 mm can be recommended to achieve an optimized perimodiolar position. A pullback of up to two marker positions bears the risk of removing the electrode tip out of its initial position.

## 1. Introduction

The position of the cochlear implant electrode inside the scalae of the cochlea has been shown to have a significant effect on the electronic compound action potential in guinea pigs [1]. The comparison of lateral and perimodiolarly positioned electrodes, even in humans, has been shown to decrease the electrical compound action potential (ECAP) thresholds [2] and the neural response telemetry (tNRT) levels [3]. In addition, a decreasing effect on the intracochlear current spread was reported [4]. The further approximation of a perimodiolar electrode by surgical modification of the insertion technique called “pullback” showed that a further focusing of the spread-of-excitation is possible [5, 6]. From a clinical perspective, the comparison of laterally positioned electrodes and perimodiolar ones showed an advantage of the latter ones in terms of frequency discrimination [7]. The direct comparison of audiological results of patients with a perimodiolar electrode and perimodiolar ones being pulled-back evidenced an increase in frequency discrimination, but not in monosyllabic understanding [8].

Since the pullback technique can be performed in different ways (e.g., modifications in insertion depth, amount of pullback) and a variability of sizes of the human cochlea [9], surgical guidelines are required. Therefore, it was the aim of the present study to estimate the change in position of perimodiolar electrodes while being pulled back in various temporal bones.

## 2. Material and Methods 

### 2.1. Preparation of TB and Surgeries

In 9 randomly chosen temporal bones, the removal of the roof of the scala vestibuli was performed. A removal of the basilar membrane was performed to obtain a panoramic view of the intra-scalar position of the array in the scala tympani. For the insertion of the electrodes (Nucleus Contour Advance array, regular electrodes), a modified round window approach was performed [10]. All insertion and pullback procedures were performed under moisturized conditions (0.9% NaCl) to simulate an in vivo intracochlear situation.

### 2.2. Estimation of the TB Size

**Figure 1:**
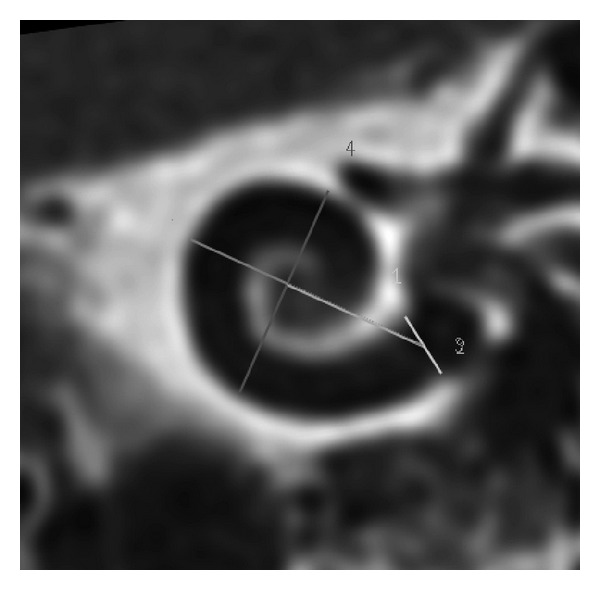
Flat-panel-based determination of cochlear size. Midth modiolar rectangular lateral wall distance (4) was used for the determination of cochlear size. (2) scalar size, (1) transmodiolar distance.

The size/dimension of the 9 temporal bones (TBs) was calculated from a flat-panel angiography scan from each temporal bone. For the measurement of the TB size, a midmodiolar lateral wall axis was used (Figure 1).

### 2.3. Pullback Procedure

For all 9 TBs encountered in the study, the pullback procedures were performed 6 times. Each contour advance electrode was used for 3 procedures. A precise description of the spatial resolution of the array position is obtained by defining the electrode markers on the array: The marker closest to the round window (RW) (when the electrode is inserted) is called number 1, the following number 2, and finally number 3. The known distance between each marker is 1 mm.

The pullback procedures performed in the TB experiments are best described by the pullback distance from marker to marker, for example, “2 → 1” (i.e., an initially inserted electrode up to position number 2 is subsequently pulled back to number 1). 

We performed the following pullback procedures in each of the 9 TBs of this study: “2 → 1,” “3 → 2,” and “3 → 1”.

 Additionally, a complete pullout was performed with different initial insertion depths, that is, insertion up to number 1 (→pullout), number 2 (→pullout), and finally number 3 (→pullout) were performed with every TB.

### 2.4. Video Capturing of the Procedures and Digital Calculations

The surgical procedures were performed under microscopic control (Moller Wedel, FS 3010, Hamburg) with an attached CCD camera (Sony IRIS) and a monitor (Panasonic TC 1470). This system was connected to a laptop. The frames were high-resolution (HR) captured by video software (Pinnacle Studio 9). The captured video was analyzed by Metra software (V 1.02). A pixel-based distance normation was performed by the known array intercontact distances and a video captured micrometer.

### 2.5. Pullback Distance Measurements

**Figure 2:**
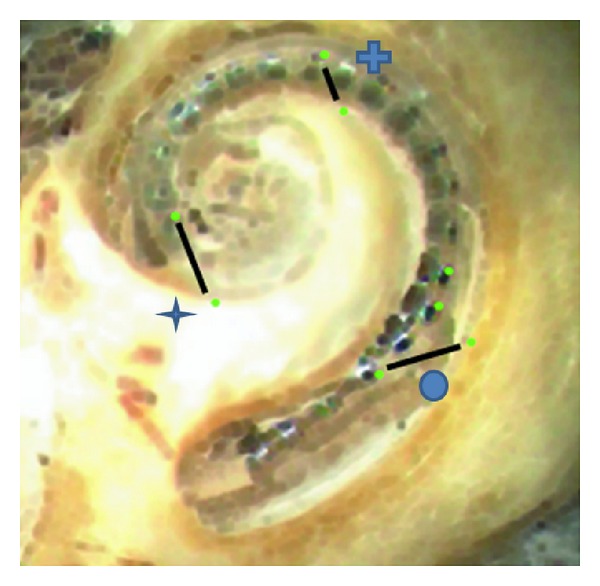
Digitally captured view on the scalar opened cochlear and the CI array with estimated distances. Tip distance (star), approximation distance (plus), and pullback distance (ball).

The software allowed the marking of certain identification points on the electrode array and the temporal bone. The marking of a point on the moving electrode and the fixed temporal bone allowed the pixel-based calculation of distances which could be transferred into mm distances by the previously performed intercontact normation. Three distances were analyzed by the software:

- a tip movement distance,
- a modiolus approximation distance,
- a pullback distance (Figure 2).

**Figure 3:**
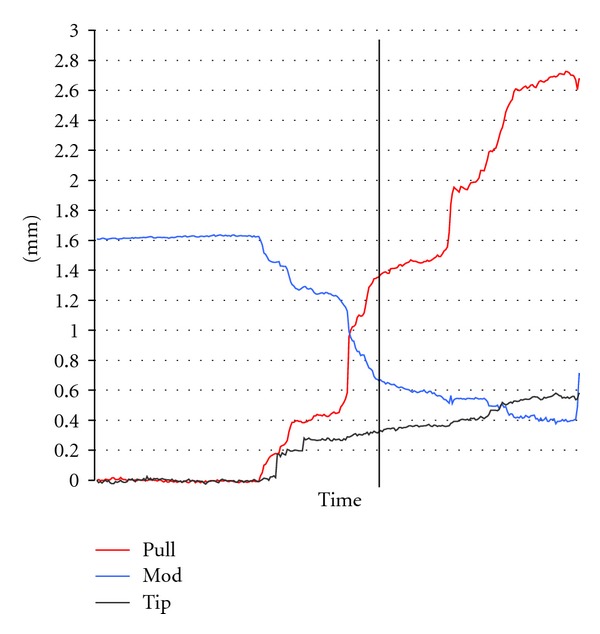
Exemplary graph of the distances estimated in a pullback procedure. Temporal changes of tip distance, approximation distance and pullback distance. The point of optimal pullback amount is marked by a vertical line.

The parallel calculation of the distances allowed a graphical description of the intracochlear position of the electrode while being pulled back over time (Figure 3). This graphical description allows the determination of an optimum pullback distance. The latter can be defined as the pullback distance without (or with limited) tip movement and decreased modiolus distance (Figure 3).

The study was approved by the Institutional Review Board (UKB, Center of Technological Research).

## 3. Results 

**Figure 4:**
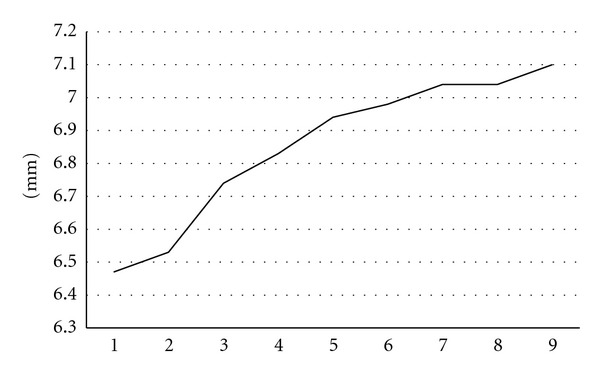
Size order of midth modiolar lateral wall distance of used temporal bones.

The midmodiolar distance was found to be variable in the temporal bones under investigation (Figure 1). We found a mean distance of 6.85 mm (Figure 4). 

The overall effect of a 2 → 1 pull back showed an intracochlearly measured pullback of the electrode of a known extent. In this approach, no tip movement was recorded. In the 3 → 1 pullback procedures, a tip movement could be observed in three out of 9 procedures. No tip movement was found in the 3 → 2 pullbacks.

There was a good correlation between the visually controlled and performed pullback and the known electrode marker distances (Table 1).

The optimum pullback distance was determined by taking into account the different initial insertion depths (as known due to the marker position at the round window and the complete pull-out of the electrode).

The following distances could be calculated as based on this rationale:

- The initial insertion to number 1 resulted in an optimum pullback distance of 1.47 mm (±0.10) with a minimal distance of 1.37 mm.
- The initial insertion to number 2 resulted in an optimum pullback distance of 2.13 mm (±0.45) with a minimal distance of 1.5 mm.
- The initial insertion to number 3 resulted in an optimum pullback distance of 2.49 mm (±0.17) with a minimal distance of 2.3 mm.

**Figure 5:**
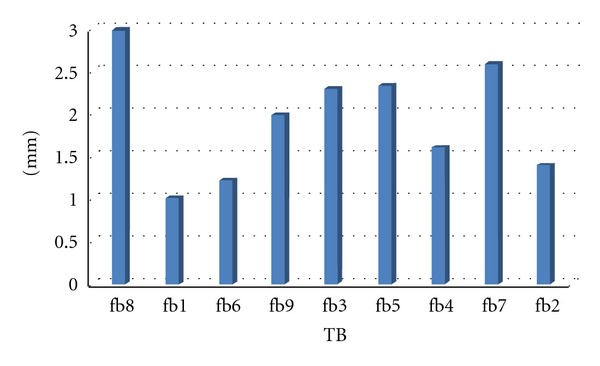
Pullback amount at the 3-1 condition in size-ordered temporal bones.

A statistically significant correlation between the different temporal bone sizes and the absolute distances of pullback (WIN-Stat, Spearmans) could not be found for the defined pullback series (Figure 5) or for the different initial ring insertions and pullbacks.

## 4. Discussion

Perimodiolar CI electrodes are assumed to offer a better frequency resolution and improved transfer of the electrical stimuli to the neural structures of the VIIIth nerve endings [11]. Wackym et al. [12] demonstrated that an improved proximity of the electrodes to the spiral ganglion cells had a positive impact on the electrical auditory brainstem response (eABR) in cats and humans for the—at that time—two different, commercially available perimodiolar electrodes. Some authors observed a decrease of the *t*-NRT levels by electrode approximation [3], while others did not [4]. An important clinical advantage of perimodiolar electrodes seems to be an increase in pitch perception [8]. 

The effect of a pullback of perimodiolar electrodes has been shown to be a focusing of the spread of excitation for the Nucleus Contour Advance [5, 6] and the Advanced Bionics Helix electrode [13]. Additionally, an increase of frequency discrimination with the Nucleus Advance array could be demonstrated after pullback [8]. A temporal bone study reported recently that the surgical technique is atraumatic for intracochlear structures [14]. However, a defined guideline for a structured procedure of this technique was still lacking. 

The comparison of the visually controlled and performed pullback orientated at the electrode markers at the round window with the video-based measured amount of pullback showed a good relation of this distance. This finding indicates a good visual control of the procedure.

An unintended tip movement was only found in three of the 3 → 1 pullbacks, but not in 2 → 1 or 3 → 2 pullbacks. Therefore, a 3 → 1 pullback cannot be recommended.

The comparison of the optimum pullback distances showed an increase at number 1 and number 2 insertions of about 0.7 mm between number 1 and number 2. This is almost the distance between the electrode markers. The difference between the number 2 and number 3 insertion is about 0.35 mm, supposedly due to an “overinsertion”. Based on these data, the optimum pullback distance should be 1.37–1.5 mm (as for number 1 and number 2) in our view. 

The known variations of temporal bone size were taken into account in this study by initial radiological calculations of cochlear size. The lack of correlation between the size of the cochleae and the pullback could be based on the repetitive use of the electrodes as well as the known product-based interelectrode variability [15]. 

## 5. Conclusion

An initial insertion to the first or second electrode marker followed by a pullback of 1.37 mm to 1.5 mm can be recommended. A pullback over the distance of two markers bears the risk of moving the electrode tip out of its initial position.

## Figures and Tables

**Table 1:** 

Mean measured pullback after a 2-1 pullback: 0.956 mm ± 0.378 mm SD/Known contact distance: 1 mm	

Mean measured pull back after a 3–1 pullback: 1.93 mm ± 0.679 mm SD/Known contact distance: 2 mm	

Mean measured pull back after a 3-2 pullback: 0.856 mm ± 0.301 mm SD/Known contact distance: 1 mm	
